# Validity and reliability of the heart failure-specific health literacy scale in Turkish

**DOI:** 10.1038/s41598-024-61154-8

**Published:** 2024-05-06

**Authors:** Asli Kalkim, Emine Karaman, Oğuzhan Birdal, Pinar Tosun Taşar

**Affiliations:** 1 Department of Public Health Nursing, Ege University Faculty of Nursing, İzmir, Turkey; 2 Department of Internal Medicine Nursing, Ege University Faculty of Nursing, İzmir, Turkey; 3 Department of Cardiology, Erzurum Atatürk University Faculty of Medicine, Erzurum, Turkey; 4 Division of Geriatrics, Department of Internal Medicine, Erzurum Atatürk University Faculty of Medicine, Erzurum, Turkey

**Keywords:** Cardiovascular disease, Heart failure, Health literacy, Methodological study, Validity and reliability, Cardiology, Health care, Medical research

## Abstract

Inadequate health literacy is common among adults with HF. The disease management process in HF closely depends on health literacy. No questionnaire is used to assess health literacy among Turkish patients with heart failure. This study aimed to determine the validity and reliability of the Turkish form of the ‘Heart Failure-Specific Health Literacy Scale’. The research is a methodological study design. The study was conducted at the cardiology clinic between May and July 2021, located in the eastern part of Türkiye. The study sample consisted of 121 patients with HF. Data were collected using the Personal Information Form and the Heart Failure-Specific Health Literacy Scale. The patients’ mean age was 62.88 ± 12.55 and 66.9% were men. Based on the factor analysis, three factors with eigenvalue above 1 have been identified. These model has been determined as x^2^ = 80.209, sd = 49 and *p* = 0.003. The fit indices were as follows: x^2^/SD = 1.637; RMSEA = 0.073, GFI = 0.90, CFI = 0.94, IFI = 0.95, TLI = 0.92 and NFI = 0.87. The scale has a total Cronbach’s alpha of 0.66. With test–retest analysis, it was determined that it had a good, positive and significant correlation in terms of both the scale and its sub-dimensions. The Turkish form of the form is a valid and reliable tool.

## Introduction

Cardiovascular diseases (CVD) are one of the major health problems worldwide. According to the World Health Organisation (WHO) report, they are the leading cause of all deaths worldwide each year^1^. In fact, it is known that by changing modifiable risk factors, the risk of cardiovascular diseases can be reduced and thus more than half of the deaths and disabilities caused by cardiovascular diseases can be reduced. However, deaths and disabilities caused by cardiovascular diseases are increasing day by day due to the inability of individuals or patients to perform healthy lifestyle behaviors^2^^.^ At the same time, Despite advances in the treatment of heart failure (HF), HF is one of the cardiovascular diseases with a high rate of morbidity and mortality^3^.

As with all chronic diseases, lifelong management of HF is important. Many of the adverse effects of HF are associated with a lack of self-care. In chronic diseases, individuals are expected to voluntarily adopt healthier behaviours and take responsibility for managing their diseases^4^. At this point, the concept of health literacy, which is effective in accessing information, comprehending, evaluating and putting it into behaviour, is important for individuals to manage their diseases effectively^5^. Considering the world population in general, health literacy levels were stated to be at low levels, which is considered as a global problem^6,7^.Inadequate health literacy is defined as individuals' limited ability to obtain, interpret, and understand basic information and health services necessary to make appropriate health decisions. Low levels of health literacy are associated with poor understanding of health information, medical education, and adherence to instructions, less engagement in preventive behaviors, delayed detection of diseases, inability to practice self-care skills, and failure to adhere to healthy lifestyle behaviors^8^.

A meta-analysis showed that inadequate health literacy is common among adults with HF and is associated with mortality and hospitalisation^9^. Additionally, it has been emphasised that the disease management process in HF depends on both literacy and health literacy/mathematical ability and that low literacy is closely associated with inadequate self-care in HF^10^. As can be seen, individuals with HF can benefit from appropriate and sufficient health literacy to manage their disease effectively. Assessing the health literacy of individuals with HF is the first step in improving this area. No Turkish form was found in the literature that assesses health literacy specifically for individuals with HF. To assess the health literacy levels of these individuals and to provide evidence-based data, valid and reliable data collection tools are necessary for the process of planning health educational programmes for individuals with HF, which is becoming more common across the country. In this direction, the study aims to determine the validity and reliability of the Turkish form of the ‘Heart Failure-Specific Health Literacy Scale’.

## Method

### Study design

To assess the psychometric properties of the Turkish version of the Heart Failure-Specific Health Literacy Scale, a methodological study design was used.

### Setting and participants

The research was conducted at the cardiology clinic between May and July 2021, located in the eastern part of Türkiye. In instrument testing, experts recommend including 10 people for every item on the instrument^11^. The study sample consisted of 121 patients to test the reliability and validity of the scale, which consists of 12 items. The inclusion criteria for patients with HF included the following: (1) aged 18 years or older; (2) speaks and understands Turkish; (3) diagnosed with HF diagnosis for at least 6 months; and (4) willingness to participate in the research. The exclusion criteria were the following: (1) individuals who are illiterate and do not want to participate in the study.

### Data collection and instruments

Data were collected using the Personal Information Form and the online form of the Heart Failure-Specific Health Literacy Scale. The data was collected via Google Forms from patients with HF registered to the cardiology service of a university hospital. For the test–retest administration, the scale was filled in again through a telephone interview with 30 patients two weeks after the first administration.

The datasets used and/or analysed during the current study available from the corresponding author on reasonable request.

- *Personal information form*: It consisted of seven questions to determine identifying characteristics of the participants including age, sex, employment status, marital status, educational status, number of individuals living together and where they live.
- *Heart failure-specific health literacy scale*: The scale was developed by Matsuoka et al. (2016)^12^. To measure the health literacy of patients with HF. This scale includes 12 items that are rated on a four-point Likert-type scale, from 1 (I completely agree) to 4 (I do not agree at all). The scale consists of three sub-dimensions: Functional HL dimension measures ‘the ability to read and write’, communicative HL measures ‘the ability to gather and transmit information’ and critical HL measures ‘the ability to critically examine information’. The scale includes a total score and scores in three sub-dimensions: functional (i.e. items 1–4), communicative (i.e. items 5–8) and critical (i.e. items 9–12). Items between 1 and 4 on the scale are scored inversely. The total score is calculated by summing the item totals. A higher score indicates a better health literacy level. The reliability of the original scale ranges from a Cronbach’s alpha of 0.71, 0.73, 0.68 to 0.69 for total HL, functional HL, communicative HL and critical HL, respectively^12^.

## Data analysis

Data were analysed using SPSS Version 24.0 and AMOS Version 25.0 (SPSS Inc., Chicago, IL). Demographic data were analysed with percentage and average. Content validity index, explanatory and confirmatory factor analyses have been used for validity. The content validity of the instrument was assessed based on the content validity index (CVI), while the construct validity was examined through an exploratory factor analysis (EFA) and CFA. To ensure data adequacy, the Kaiser–Meyer–Olkin (KMO) test and the Bartlett’s test of sphericity were used for the factor analyses. As with the original scale, the Maximum Likelihood method and the promax rotation technique were used for the EFA. CFA was performed to evaluate the factor structure for adequate model fit with multiple fit indices. Multicollinearity has been assessed, and the items’ VIF and tolerance values have been analysed for confirmatory factor analysis. Cronbach’s alpha was used to estimate the internal consistency. Item-to-total and inter-item correlations were estimated using Pearson’s correlation coefficient. Test–retest analyses and intraclass correlation calculation (ICC) have been used. Response bias in the scale has been analysed through the Hotelling T2 test. The scale’s collectability has been assessed through the Tukey collectability analysis. All tests were performed at a statistical significance level of *p* < 0.05.

## Procedures

### Language validity

For language validity, two English linguists whose native language is Turkish independently translated the scale from English to Turkish. Later, the researchers evaluated the most accurate translation for each item and developed a joint Turkish text. The scale translated into Turkish was compared with its original form by the retranslation method after being translated back into English by two linguists who are fluent in both Turkish and English. Finally, inappropriate terms were reviewed and language validity was ensured.

### Content validity

The draft scale was submitted to 10 experts (2 cardiologists, 3 clinical nurse specialising in cardiovascular nursing, 4 nurse scientists and 1 clinical psychologists) to assess the content validity of the measures. The experts evaluated the items’ language and content. To assess content validity, the scale items were scored between 1 (not appropriate) and 4 (completely appropriate). Points were assessed using the scope of the validity index.

### Preliminary stage

Based on expert opinions, the draft was revised and was administered to 20 nonparticipants who complied with the sample characteristics. The scale was then decided to be applied to a larger group since no negative feedback was received regarding the items’ clarity.

### Ethical considerations

Permission to use the English version of the scale was obtained from the original authors via e-mail. This study complied ethically with the declaration of Helsinki and was approved by the Ege University Institutional Review Board in İzmir, Türkiye (IRB No: 21–11.1 T/54). A written informed consent was obtained from all study participants. The details disclosed included the study purpose, confidentiality, participants’ autonomy, voluntary participation and freedom to withdraw from participating at any time. All of the participants completed the questionnaires for approximately 10–15 min.

## Results

### Descriptive sample characteristics

The study participants’ mean age was 62.88 ± 12.55 (min, 23; max, 86); 66.9% were men, 46.3% of them were primary school graduates and 86.0% were married.

### Validity analysis

#### Content validity

Following the review of the scales’ items by ten experts, some items were re-evaluated, no items were removed from the scale and the I-CVI ranged from 0.89 to 1.00 and the S-CVI was 0.94.

#### Construct validity

Bartlett’s test of sphericity showed that Kaiser–Meyer–Olkin (KMO) was 0.772 and x^2^ = 593.755, df = 66 and *p* = 0.000. Based on the factor analysis, three factors with eigenvalue above 1 have been identified. Table 1 shows the eigenvalue and variance explained by the factors.

**Table 1:** The explanatory factor analysis results of the turkish version of the heart failure-specific health literacy scale.

Items	Factor loading		
	Factor 1	Factor 2	Factor 3
Item 1	0.660		
Item 2	0.838		
Item 3	0.861		
Item 4	0.730		
Item 5		0.743	
Item 6		0.964	
Item 7		0.614	
Item 8		0.261	0.511
Item 9			0.333
Item 10			0.436
Item 11			0.863
Item 12			0.550
Eigenvalue	3.936	2.476	1.398
Explained variance (%)	29.211	17.612	6.815
Total explained variance (%)	53.637		
KMO	0.772		
Bartlett x^2^ (p)	593.755 (0.000)		

The first, second and third factors explain 29.2%, 17.6% and 6.8% of the total variance, respectively. These factors explained 53.6% of the total variance. The factor loading of the first, second and third factors ranged from 0.660–0.861, 0.261–0.964 and 0.333–0.863, respectively (Table 1).

The three-factor model has been determined as x^2^ = 80.209, sd = 49 and *p* = 0.003. The fit indices were as follows: x^2^/SD = 1.637; RMSEA = 0.073, GFI = 0.90, CFI = 0.94, IFI = 0.95, TLI = 0.92 and NFI = 0.87 (Table 2).

**Table 2:** Model fit indices of the scale.

	x^2^	SD	x^2^/SD	RMSEA	GFI	CFI	IFI	NFI	TLI
Three-factor model	80.209	49	1.637	0.073	0.90	0.94	0.95	0.87	0.92

*χ2*, Chi-square; *RMSEA*, root mean square error of approximation; *GFI*, goodness-of-fit index; *CFI*, comparative fit index; *IFI*, incremental fit index;* NFI*, normed fit index;* TLI*, Tucker–Lewis Index.

**Figure 1:**
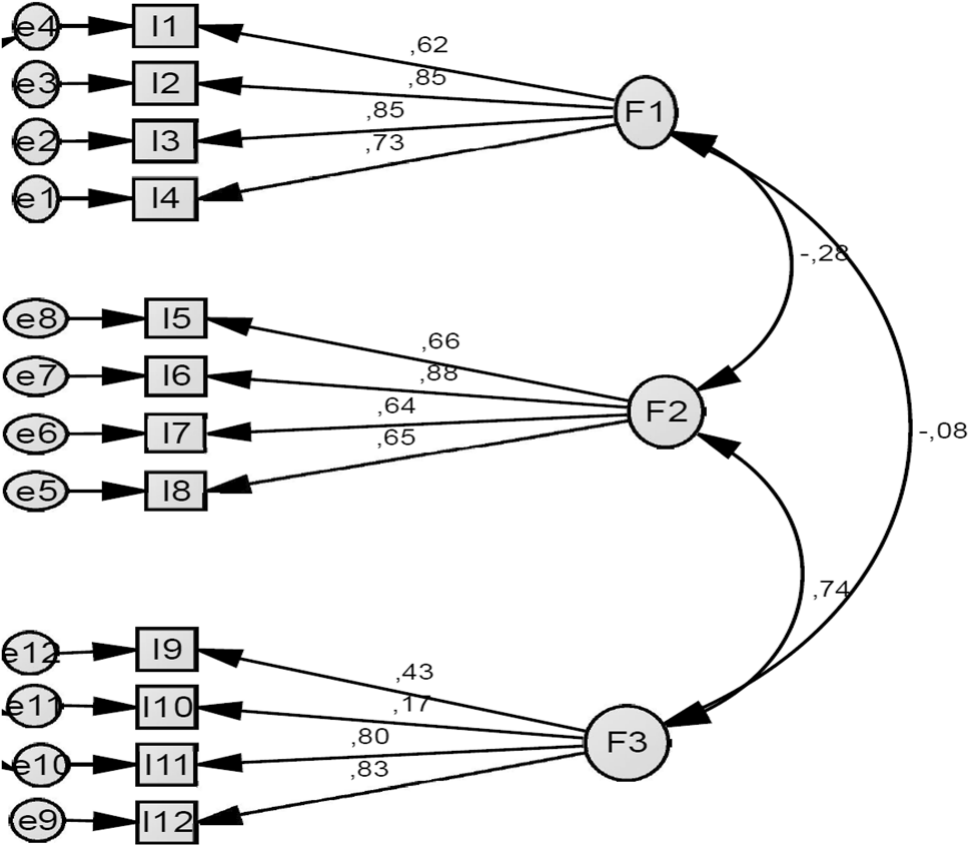
Confirmatory factor analysis of the heart failure-specific health literacy scale.

The CFA results indicated that factor loading of the first sub-dimension ranged between 0.62 and 0.85, factor loadings of the second sub-dimension ranged between 0.66 and 0.88 and factor loadings of the third sub-dimension ranged between 0.17 and 0.83 (Fig. 1).

#### Reliability analysis

**Figure 2:**
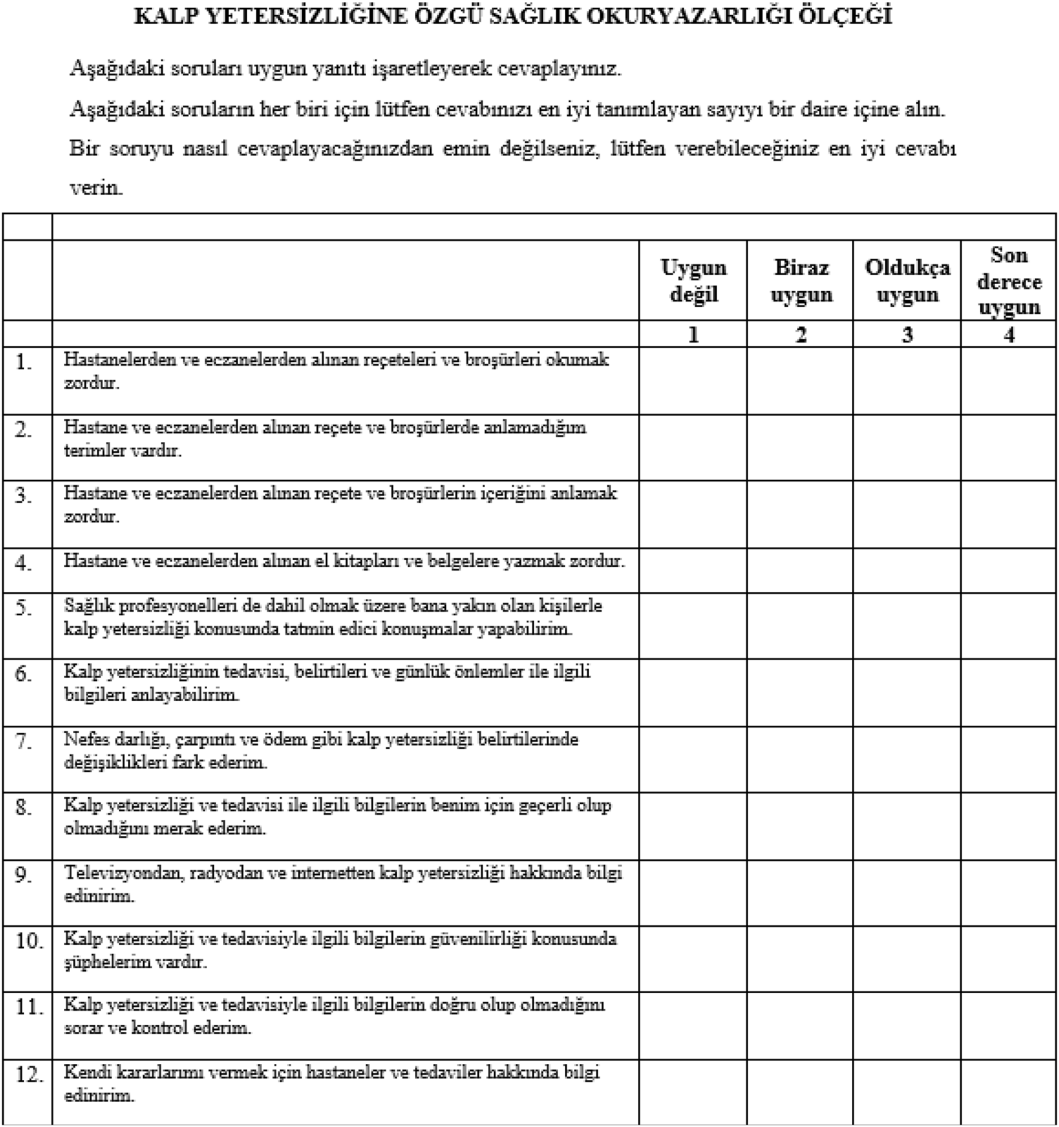
Turkish form of the ‘heart failure-specific health literacy scale’.

The scale has a total Cronbach’s alpha of 0.66. The first, second and third sub-dimensions have a Cronbach’s alpha of 0.85, 0.79 and 0.67, respectively. The correlations of the scale items with the scale total score were found in the range of 0.320–0.642, and the corrected item-sub-dimension total score correlation ranged between 0.276 and 0.750 (Table 3). The Turkish scale’s Hotelling T-square value was determined to be 257.184, F = 21.432 and *p* = 0.000. In Tukey additivity analysis, F = 0.018 and *p* = 0.892 (Fig. 2).

**Table 3:** Reliability of the HF-Specific HL Turkish Version (n = 121).

Items	Total scale cronbach’s alpha	Sub-dimensions’ cronbach’s alpha	Item–total score correlations (r)*	Item–sub-dimensions total score correlations (r)*
Item 1	0.66	0.85	0.320	0.571
Item 2			0.370	0.749
Item 3			0.496	0.745
Item 4			0.399	0.667
Item 5		0.79	0.461	0.597
Item 6			0.536	0.750
Item 7			0.409	0.571
Item 8			0.468	0.497
Item 9		0.67	0.529	0.431
Item 10			0.330	0.276
Item 11			0.617	0.539
Item 12			0.642	0.572

*p < .001.

Test–retest analysis showed that no statistically significant difference was found between the test–retest mean scores of the second and third sub-dimensions of the scale (*p* > 0.05). However, a statistically significant difference was found between the test–retest mean scores of the first sub-dimension of the scale (*p* < 0.05). The two measurements were determined to have a good correlation, positive and significant for both the scale and its sub-dimensions (*p* < 0.05) (Table 4). The ICC calculated for the two measurements were determined to be 0.729.

**Table 4:** Test–retest reliability analysis of the HF-specific HL Turkish version (n = 121).

Scale and sub-scales	Test M ± SD	Retest M ± SD	t *p*	r *p*
HF-specific HL	33.33 ± 6.72	32.06 ± 4.12	1.357 0.185	0.650 0.000
Functional HL	9.26 ± 3.47	7.96 ± 2.95	2.258 0.032	0.529 0.003
Communicative HL	12.66 ± 3.04	12.96 ± 2.63	0.529 0.601	0.407 0.025
Critical HL	11.40 ± 3.10	11.13 ± 2.43	0.510 0.614	0.486 0.006

## Discussion

This research aimed to evaluate the validity and reliability of the Turkish form of the Heart Failure-Specific Health Literacy Scale. The scale, with its three sub-scales and 12 items, was found to be a valid and reliable tool to be used in the Turkish culture.

The I-CVI and S-CVI values of the Turkish version of the scale were higher than 0.80. This result indicated that the items in the scale adequately represented the characteristics to be measured regarding quantity and quality^13–17^. In the studies conducted with other versions of the scale, CVI value in Persian and Chinese versions was found to be higher than the recommended value. However, the CVI value was observed to have not been evaluated in the original scale study.

The KMO values were classified as excellent (1.00–0.90), very good (0.89–0.80), good (0.79–0.70), moderate (0.69–0.60), poor (0.59–0.50) and insufficient when it is below 0.50. In this study, since the KMO value of the scale was higher than 0.70, the sample size for factor analysis was considered to be at a good level^12^ Turkey. (2016).

The eigenvalue must be ≥ 1 to determine the number of factors^20^. In this study, the scale was observed to have three sub-dimensions. The three sub-dimensions explained 53.6% of the total variance. The literature shows that variance ratios explained between 50 and 60% are generally quite high^18,23^. In the study with the original scale, the three-factor scale explained 55.95% of the variance. The validity and reliability study using the scale’s Chinese version found that the three-factor structure explained 64.62% of the total variance^24^ . The three-factor structure has been found in the scale’s Persian version^25^. In order for patients with HF to change their daily lives and evaluate the signs and symptoms related to their disease, appropriate information should be obtained and self-care behaviours should be used effectively. Therefore, evaluating the functional HL alone is insufficient. In the Turkish version of the scale, the first, second and third sub-dimensions were named as functional, communicative and critical HL, respectively. The three-dimensional structure of the scale will contribute to the evaluation of the patients’ HL from a multidimensional perspective^12^.

As a result of EFA, the factor loadings of the three-factor scale were > 0.30 as recommended by the literature, except for the 8th item^18,26,27^.

Item 8 ‘I wonder if the information about HF and its treatment applies to me’ has a factor loading of 0.26. In the original scale, this item had a factor loading of 0.61. In the Persian version, the factor loadings of all items ranged between 0.56 and 0.88, and the factor loading of item 8 was the lowest compared to the other items (0.56)^24^. In the Chinese version, the factor loading of this item was 0.553 and was lower than other items^24^. The eight items in the original and other versions were not removed from the scale, and the number of items remained 12 as in the original scale.

Based on the CFA analysis results, five of the seven fit indices (RMSEA, NFI, GFI, IFI and CFI) showed good fit and two (× 2/df, TLI) showed excellent fit^28^. CFA was not performed in the original study. The scale’s Chinese version showed that five of the six fit indices have an acceptable fit.

The correlation value should be > 0.20, positive and as close to 1 as possible^27^. This study showed the correlation value of each item with the total score on the scale in ranged between 0.32 and 0.64. Moreover, the item–total score correlation coefficients were positive and > 0.20, and no item was removed from the scale. Hence, all items in the scale showed a high correlation with the total score, the scale measured the desired quality sufficiently and the scale’s item reliability was high.

In this study, as a result of the reliability analysis of the Turkish version of the scale, the Cronbach’s alpha coefficients of the whole scale, the second sub-dimension and the third sub-dimension were between 0.60 and 0.79, so it was considered highly reliable. The Cronbach’s alpha value of the first sub-dimension was between 0.80 and 1.00, so it was also considered highly reliable^14^. The original scale had a Cronbach’s alpha of 0.71, 0.73, 0.68 and 0.69 for total HL, functional HL, communicative HL and critical HL, respectively^12^. The scale’s Chinese version had Cronbach’s alpha values of 0.87 for the overall scale and 0.84, 0.72 and 0.69 for the three dimensions, respectively, indicating acceptable internal consistency^24^. The Persian version of the scale had a Cronbach’s alpha values higher than 0.70^25^.

The test–retest results of the scale and its sub-dimensions showed that the scale was invariant over time and with consistent results. The original scale also indicated that the test–retest result had a good test–retest reliability^10^. Test–retest evaluation of the scale’s Persian version showed that the stability of the scale was satisfactory to good^25^.

## Conclusion

In conclusion, study findings showed that Turkish form of the Heart Failure-Specific Health Literacy Scale is a valid and reliable tool. The scale is easy to use and is recommended to be used as a screening tool in various settings including the hospital, clinic or health centre to identify HF patients with low HL levels. The scale can be utilised in future research and nursing practice to improve the health literacy of HF patients and to guide them in doing self-care.

## Limitations

The study limitation is that the sample of the study consists of patients receiving health services from only one centre.

## References

[1] WHO. Cardiovascular diseases (2021).

[2] Raesi R (2023). Risk factors of acute coronary syndrome: the experience from Iran. Open Public Health J..

[3] McDonagh TA (2021). Corrigendum to: 2021 ESC Guidelines for the diagnosis and treatment of acute and chronic heart failure: developed by the task force for the diagnosis and treatment of acute and chronic heart failure of the european society of cardiology (ESC) With the special contribution of the heart failure association (HFA) of the ESC. Eur. Heart J..

[4] Mackey LM, Doody C, Werner EL, Fullen B (2016). Self-management skills in chronic disease management: what role does health literacy have?. Med. Decis. Making.

[5] Guntzviller LM, King AJ, Jensen JD, Davis LA (2017). Self-efficacy, health literacy, and nutrition and exercise behaviors in a low-income, hispanic population. J. Immigr. Minor. Health.

[6] Liu H (2018). Assessment Tools for health literacy among the general population: a systematic review. Int. J. Environ. Res. Public Health.

[7] Özdemir S, Akça HŞ, Algın A, Kokulu K (2020). Health literacy in the emergency department: a cross-sectional descriptive study. Eur. J. Emerg. Med..

[8] Raesi R (2022). Assessment of health literacy and self-care behaviors among patients discharged from covid-19 wards. Arch. Adv. Biosci..

[9] Peberdy MA (2010). Part 9: post-cardiac arrest care: 2010 american heart association guidelines for cardiopulmonary resuscitation and emergency cardiovascular care. Circulation.

[10] Chobanian AV (2003). Seventh report of the joint national committee on prevention, detection, evaluation, and treatment of high blood pressure. Hypertension.

[11] Aksayan S, Gözüm S (2002). Guide for transcultural adaptation of the scale II: psychometric characteristics and cross cultural comparison. Hemşirelikte Araştırma Geliştirme Dergisi.

[12] Matsuoka S (2016). Development and Validation of a heart failure-specific health literacy scale. J. Cardiovasc. Nurs..

[13] Alpar R (2018). Applied statistics and validity-reliability with examples from sports, health and educational sciences.

[14] Burns, N. & Grove, S. in *The practice of nursing research* (Philadelphia, PA: W. B. Saunders, 2009).

[15] Buyukozturk S (2012). Data analysis hand book for social sciences.

[16] Rubio DM, Berg-Weger M, Tebb SS, Lee ES, Rauch S (2003). Objectifying content validity: conducting a content validity study in social work research. Social Work Res..

[17] Yurdugul, H. ( Ulusal Eğitim Bilimleri Kongresi, Denizli, 2005).

[18] DeVellis R (2012). Scale development.

[19] Hayran, M. & Hayran, M. *Basic statistic for health research*. (Art Ofset Maatbacılık Yayıncılık,Ankara, 2011).

[20] Jonhson B, Christensen L (2014). Educational research: quantitative, qualitative, and mixed approaches.

[21] Kalaycı, S. *SPSS Applied Multivariate Statistics Techniques*. (BRC Printing, Ankara, Turkey, 2016).

[22] Field A (2018). Discovering statistics using IBM SPSS statistics.

[23] Balci, A. *Research methods, techniques, and principles in social sciences* 9th edn, (Pegem Academy Publishing, 2011).

[24] Yue M, Zhang L, Lu Y, Jin C (2016). Translation and psychometric evaluation of the Chinese version of the heart failure-specific health literacy scale. Int. J. Nurs. Sci..

[25] Barati M, Taheri-Kharameh Z, Farghadani Z, Rasky E (2019). Validity and reliability evaluation of the persian version of the heart failure-specific health literacy scale. Int. J. Commun. Based Nurs Midwifery.

[26] Polit D, Beck C (2017). Nursing research: generating and assessing evidence for nursing practice.

[27] Sencan H (2005). Reliability and validity in social and behavioral measures.

[28] Vieira A (2011). Interactive LISREL in Practice: Getting Started with a SIMPLIS Approach.

